# Kissing molars class III detected at a young age

**DOI:** 10.1186/s40902-023-00388-4

**Published:** 2023-05-23

**Authors:** Teruhide Hoshino, Yu Koyama, Akira Katakura

**Affiliations:** Department of Oral Pathobiological Science and Surgery, Tokyo Dental College, Chiyoda-Ku, Tokyo, 101-0061 Japan

**Keywords:** Kissing molars, Wisdom teeth, Impacted teeth, Young age

## Abstract

**Background:**

Kissing molars (KMs) is defined as a state in which the apex of two impacted molars face opposite directions and the occlusal surfaces touch each other and the crown is in one follicle. Class III KMs have been reported previously; however, reports on class III KMs in young people (< 18 years of age) are limited.

**Case presentation:**

Here, we present the case of KMs class III confirmed at an early age, supported by a review of the literature. The patient was a 16-year-old female and experienced discomfort in the left molar of the lower jaw and visited in our department. We diagnosed KMs based on an impacted teeth on the buccal side, near the lower jaw wisdom teeth, and a cyst-like low-density area observed around the crown of both teeth, as revealed by computed tomography. We decide to extract the tooth and enucleate the cyst under local anesthesia as the patient experienced discomfort due to occlusion. Furthermore, the cyst-like structure removal and tooth extraction including tooth root were necessary as the patient had KM class III, possibly inducing complicated malocclusion. Although no previous reports recommended timing for KMs tooth extraction, we propose that extraction at an early stage is important regardless of age especially in class III cases.

**Conclusions:**

We report a case of KM class III detected at an early age.

## Background

Kissing molars (KMs) is defined as a state in which the apex of two impacted molars face opposite directions and the occlusal surfaces touch each other and the crown is in one follicle [1]. Class III KMs have been reported previously; however, reports on class III KMs in young people (< 18 years of age) are limited. Moreover, KMs have been proposed to be associated with delayed tooth eruption, odontogenic cysts [2, 3], and mucopolysaccharidosis [4]. However, the mechanism of formation of KMs is not completely understood. Therefore, in this report, we present the case of a 16-year-old woman with class III KMs.

## Case presentation

**Fig. 1:**
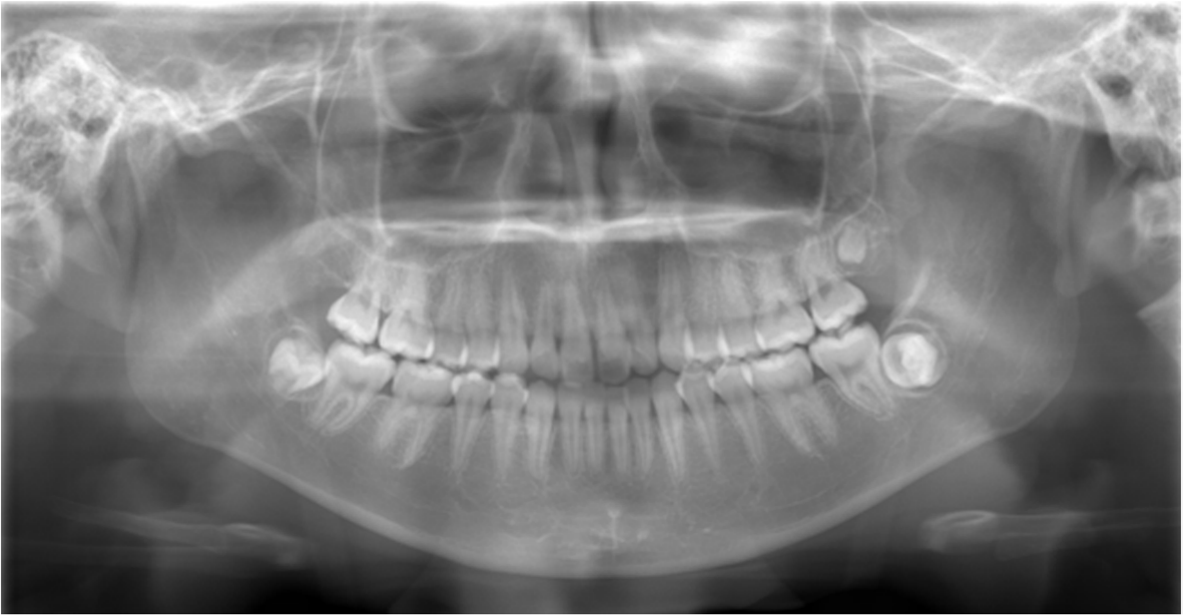
Panoramic X-ray at the first visit. The left mandibular wisdom tooth was impacted, and a further permeation zone was observed around the crown

A 16-year-old female presented with discomfort due to occlusion of the lower jaw left side on January 2022. She and her family had no medical history of complications. In January 2022, after examination at a dental clinic, the patient was referred to our department in Tokyo. Oral findings revealed mild swelling in the buccal gingiva of the second mandibular molar, and no inflammatory findings were observed in the gingiva. Panoramic radiography revealed that the left wisdom tooth of the lower jaw was impacted horizontally, and a further transmission zone was observed around the crown (Fig. 1).

**Fig. 2:**
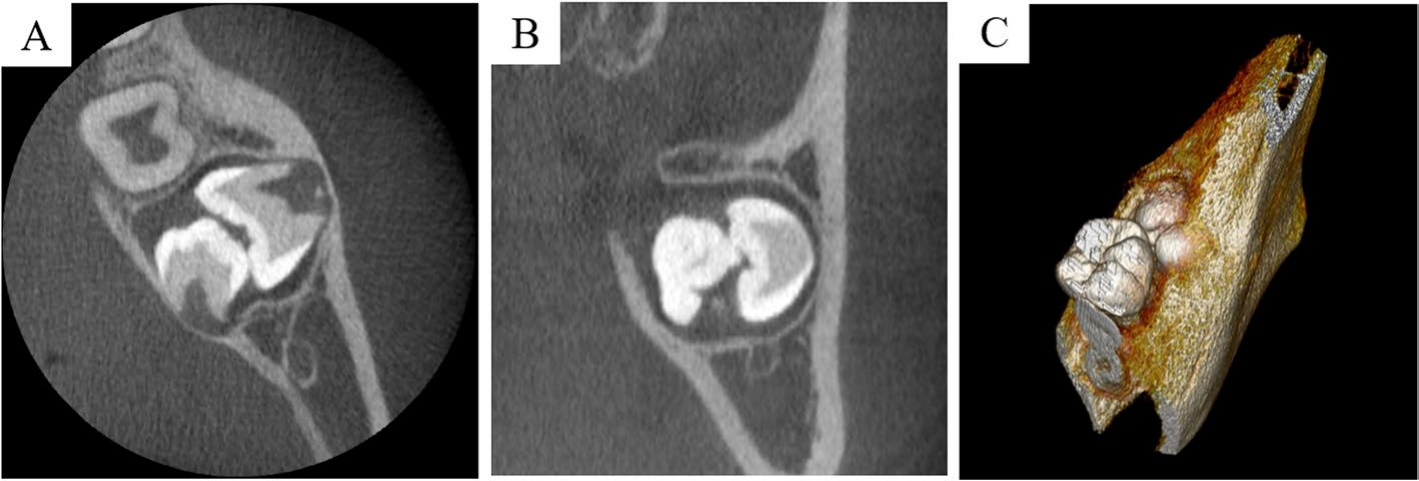
Computed tomography image (**A** horizontal section, **B** coronal section, **C** 3D construction). Two impacted teeth located on the buccal tongue side were observed in one dental sac of the left side of the lower jaw, and a cyst-like low-density area was observed around the crown of the two teeth

Computed tomography (CT) revealed two impacted teeth located on the buccal-lingual side in one dental sac of the left lower jaw and a cyst-like low-density area around the crown of the two teeth (Fig. 2A, B, C). Thus, we diagnosed the patient with KMs class III.

**Fig. 3:**
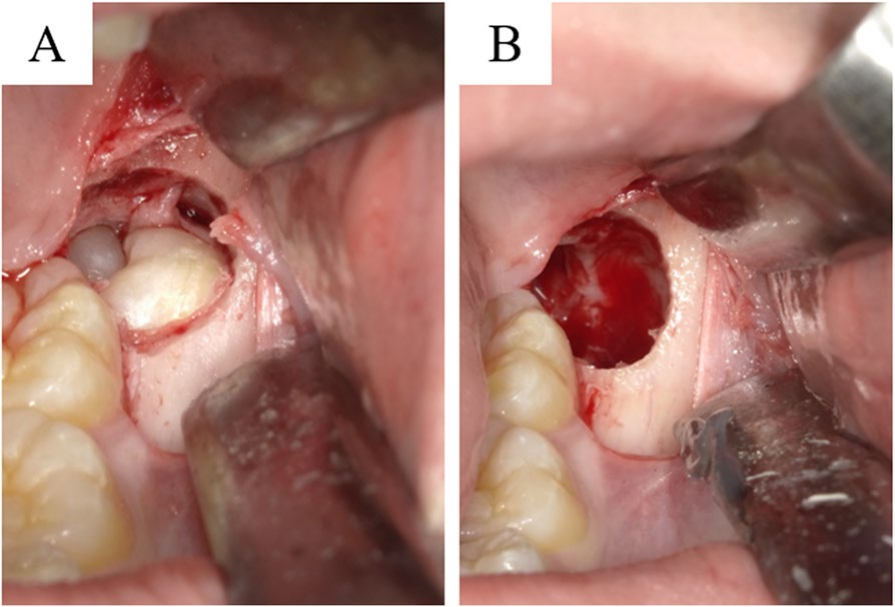
Intraoperative photograph. **A** Manifestation of crown. The crowns of the teeth face each other, and the occlusal surfaces are in contact with each other. **B** After tooth extraction. The crown division was performed to extract the tooth and remove the cyst wall

**Fig. 4:**
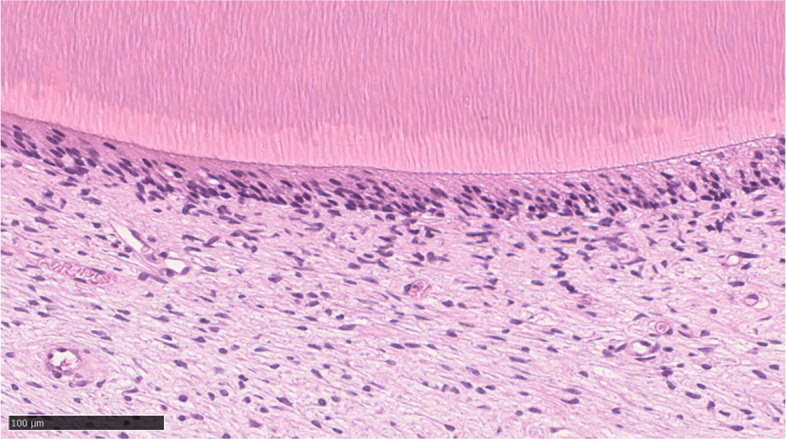
Histopathological staining. H-E staining of the tissue around the tooth revealed connective tissue with a tendency to regressive enamel epithelium and fibrosis

Two months after the first visit, impacted tooth removal and enucleating of cyst were performed under local anesthesia. Neumann incision was made between distal of the left second molar and mesial-buccal side in mandibular, and the mucoperiosteal flap was reversed to remove the buccal cortex bone (Fig. 3A). First, the crown of the excess tooth and that of the third molar were divided and removed. Then, the cyst wall was removed with the crown removal (Fig. 3B). Subsequently, the wound was completely closed. No intraoperative or postoperative incident was observed; the discomfort reported at the first visit disappeared 1 year after the operation, and the course was good. Histopathological findings revealed that the tissue around the tooth was connective tissue with enamel epithelium regression tendency (Fig. 4). The histopathological diagnosis was dental sac.

## Discussion

KMs have been proposed to occur due to delayed tooth eruption and odontogenic cysts [2, 3], and related to mucopolysaccharidosis [4], the mechanism of KMs development has not been completely deciphered. So far, 57 cases of KMs have been reported, of which 7 were juveniles (< 18 years old) [4–10]. All the seven cases had class I or class II KMs, the direction of the occlusal surface was near centrifugal, and the root of the tooth was complete (Table 1).

**Table 1:** KMs detected in younger patients

	Year	Gender	Age	Classification	Direction	Tooth roots	Radiolucent areas	Treatment
Nakamura et al. [4]	1992	M	17	True	ー	Present	ー	ー
				One side, class II				
Gulses et al. [6]	2012	M	16	True	Mesial-distal side	Present	Present	Extraction
				One side, class II				Enucleation
Güven et al. [7]	2013	F	13	True	Mesial-distal side	Present	ー	Extraction
				One side, class I				
Kiran et al. [8]	2014	F	18	Pseudo	Mesial-distal side	Present	Present	Extraction
				Both sides, class II				Enucleation
Nedjat-Shokouhi et al. [5]	2014	M	18	True	Mesial-distal side	Present	Present	Extraction
				One side, class II				Enucleation
Anubhav et al. [9]	2014	F	18	True	Mesial-distal side	Present	ー	Extraction
				One side, class II				
Barros et al. [10]	2018	F	10	Pseudo	Mesial-distal side	Present	Absent	Orthodontic treatment
				One side, class I				
This case	2022	F	16	True	Buccal-lingual side	Absent	Present	Extraction
				One side, class III				Enucleation

KMs are classified based impacted teeth type into class I (mandibular first molar and second molars), class II (mandibular second molar and mandibular third molars), and class III (mandibular third molar and mandibular fourth molars [11]. Nedjat-Shokouhi et al. [5] and Menditti et al. [12] classified KMs with contact between occlusal surfaces as true KMs and those without occlusal surface contacts as pseudo KMs. They further distinguished KMs into cystic variants with cyst-like transmission images and those without cystic variants without cyst-like transmission images on X-ray images. Moreover, the fourth molars impaction, defined as class 3, is the fourth most common after maxillary median, maxillary fourth molars, and maxillary lateral incisors [13, 14]. Class III KMs are further classified into molars and posterior teeth KMs based on their location [15]. KMs are further classified based on structure into cylindrical, nodular, and funnel-type forms [16, 17] and based on buried positions into vertical, inclined, and horizontal KMs [18].

However, evaluation of the morphology and impacted position of the tooth are difficult in people with incomplete root formation and alignment of teeth. Moreover, in class III KMs with incomplete root formation and KMs located on the buccal side, detection of impaction is difficult using panoramic X-rays.

Shahista et al. have reported the case of the youngest patient so far with class III KMs [19]. As the patient was 21 years old, we propose that class 3 KMs are rarely detected early. Moreover, young people generally do not consider wisdom tooth extraction unless symptoms appear or orthodontic treatment becomes necessary. In this case, the patient was referred from a nearby dental clinic, and an odontogenic cyst was detected based on panoramic X-ray images with no excess teeth protruding. These results suggest that in many cases, KMs are confirmed by dentists after the root formation and wisdom teeth eruption. The movement of impacted teeth within the jawbone [20] occurs in the bone marrow with little resistance during root formation as teeth move toward the crown due to the blood flow pressure through the apical foramen. Moreover, impacted teeth movement is more common in single root teeth and teeth with little root diastasis. Thus, class III KMs are impacted on the buccal side during the embryogenesis stage and move in the jawbone during teeth root formation.

In this case, the fourth molar was located on the buccal side from the impacted position, and the third molar was horizontally impacted. Additionally, the pulpal cavity morphology of the X-ray image suggests that the third molar had a single root, and the excess tooth had a compound root. Thus, with no treatment, the third molar with no single root may have moved upward in the bone marrow at the anterior edge of the mandibular branch instead of in the direction of the dense buccal shelf due to the presence of the compound root of the excess teeth. Therefore, the patient may be diagnosed with true KMs class III with cystic variant exhibiting an inclined buried position.

Treatment of KMs is often selected on class classification using X-ray images, the presence or absence of cystoid transmission image, and clinical symptoms. Tooth extraction is performed in all cases with cyst-like transmission image observed in past reports. Moreover, class III cases are difficult to induce eruption, and tooth extraction is often recommended [21]. However, class I and II cases are often juvenile. Orthodontic treatment is recommended in cases where dentition can be guided regardless of the presence or absence of cyst-like transmission image, using fenestration in combination [8].

In this case, tooth extraction was selected because of (1) awareness of discomfort during occlusion, (2) KMs being class III, (3) the necessity of removal of cyst-like structures, and (4) the possibility of complicated malocclusion and tooth extraction procedures with the completion of the tooth root formation.

The recommended timing for extraction of KMs is not reported yet. However, early tooth extraction should be considered regardless of age, especially in class III cases.

## Conclusions

In this study, we report a case of KM class III detected at a young age.

## Data Availability

The data of this case are available from the corresponding author upon reasonable request.

## Abbreviations

KM: Kissing molars
CT: Computed tomography
